# The Disability Rate of 5-Year Post-Stroke and Its Correlation Factors: A National Survey in China

**DOI:** 10.1371/journal.pone.0165341

**Published:** 2016-11-08

**Authors:** Yang Yang, Yu-Zhi Shi, Ning Zhang, Shuo Wang, Gabor S. Ungvari, Chee H. Ng, Yi-Long Wang, Xing-Quan Zhao, Yong-Jun Wang, Chun-Xue Wang, Yu-Tao Xiang

**Affiliations:** 1 Department of Neurology, Beijing Tian Tan Hospital, Capital Medical University, Beijing, China; 2 Department of Neuropsychiatry & Behavioral Neurology and Clinical Psychology, Beijing Tian Tan Hospital, Capital Medical University, Beijing, China; 3 China National Clinical Research Center for Neurological Diseases, Beijing, China; 4 Center of Stroke, Beijing Institute for Brain Disorders, Beijing, China; 5 Beijing Key Laboratory of Translational Medicine for Cerebrovascular Disease, Beijing, China; 6 University of Notre Dame Australia / Marian Centre, Perth, Australia; 7 School of Psychiatry & Clinical Neurosciences, University of Western Australia, Perth, Australia; 8 Department of Psychiatry, University of Melbourne, Melbourne, Victoria, Australia; 9 Unit of Psychiatry, Faculty of Health Sciences, University of Macau, Macao SAR, China; Massachusetts General Hospital, UNITED STATES

## Abstract

Few studies on long-term functional outcome have been conducted in post-stroke patients in China. The objective of this study was to conduct a nationwide survey in China to investigate the 5-year prevalence of post-stroke disability and its correlation factors. A total of 893 patients with ischemic stroke were included. Demographic, clinical and neuro-imaging information were collected with standardized instruments that assessed stroke severity, depression, cognitive impairment, stroke recurrence and physical disability. Disability was assessed with the modified Ranking Score (mRS), of which a cutoff score ≥2 indicates disability. Statistical analysis included chi-square tests, two independent samples t-tests, Mann-Whitney U test and multiple logistic regression analysis. The frequency of disability in this study population was 45%. Multivariate analyses revealed that older age, lower education level, previous history of stroke, stroke severity at admission, depression, cognitive impairment at 3 months, and stroke recurrence within 5 years follow up were all significantly associated with post-stroke disability. The disability rate in 5-year post-stroke was high in Chinese patients. Treatment of depression, secondary prevention of stroke and rehabilitation may benefit disabled patients with stroke in China.

## Introduction

In 2010, approximately 16.9 million persons worldwide developed first-ever stroke. There was an estimate of 33 million stroke survivors [1], of whom a substantial proportion suffered from disability. Disability after stroke is a significant outcome that leads to personal suffering and burden on their families [2]. Most studies have focused on short-term data within 3 months post-stroke [3]. However, long-term functional status, as measured by the activities of daily living (ADL) in one particular study, does not remain stable between 3 months and 1 year post-stroke [4]. Another 5-year follow-up study found a delayed but significant functional decline in stroke survivors [5]. Growing evidence supports the importance of early rehabilitation intervention after stroke, but strategies to reduce the risk of long-term post-stroke disability beyond a year remain unclear. Long-term post-stroke outcomes are usually determined by different factors compared to short-term outcomes [6, 7]. In addition, post-stroke disability significantly increases the risk for adverse outcomes, such as recurrent stroke and increased mortality [8, 9]. Thus, the identification of post-stroke disability, associated risk factors, and appropriate rehabilitation strategies are important for long-term prognosis of stroke survivors.

The objective of this study was to conduct a nationwide survey in China to investigate the 5-year prevalence of post-stroke disability and its associated risk factors.

## Methods

### Study Settings and Patients

This 5-year follow-up study of stroke patients is a national project entitled the “Prospective Cohort Study on the Incidence and Outcome of Patients with Post-stroke Depression in China (PRIOD)” [10]. At baseline, patients in 56 hospitals nationwide were consecutively recruited and enrolled if they were aged 18 years or older, had an acute stroke according to WHO criteria [11] confirmed by CT or MRI within 14 days, could complete a clinical interview and provide informed consent. Patients with severe cognitive impairment and history of drug dependence were excluded. The study protocol was approved by the medical ethics committees of Beijing Tiantan Hospital and the participating institutions, respectively. All participants provided written informed consent.

### Measurement Tools and Evaluation

In this study we used data at baseline, 3 months and 5 years after the index stroke. Basic socio-demographic, clinical and neuro-imaging features at baseline were recorded using a standard form, and supplemented by medical records review and a clinical interview by qualified neurologists.

Stroke severity was assessed at baseline by the National Institutes of Health Stroke Scale (NIHSS) [12]. Stroke lesions was classified as left, right, frontal, temporal, occipital-parietal lobes, basal ganglia, thalamus, brainstem and cerebellum based on MRI or CT scans reported by radiologists. Current smoking was defined as at least 1 cigarette in the past month. Moderate/heavy drinking was defined as ≥2 standard drinks/day.

Post-stroke depression at 3 months follow up was based on the checklist of the DSM-IV criteria [13]. The Chinese version of the Hamilton Rating Scale for Depression (HRSD) [14, 15] was used to assess severity of depression. Cognition function was measured by the Mini Mental State Examination (MMSE) [16] at 3 months after stroke. Cognitive impairment was defined as the MMSE total score<24 [17, 18].

The 5-year assessments including stroke recurrence and physical disability were conducted via a telephone interview. Recurrent stroke was defined as any type of stroke diagnosed at re-admission during the past 5 years [19]. Post-stroke functional status was assessed using the modified Ranking Score (mRS), with a score ≥2 as meeting the criteria for disability [20].

### Statistical analyses

Statistical analyses were performed with SPSS Statistics 21.0 for Windows. The comparisons between the disability and non-disability groups with regard to demographic, clinical and neuro-imaging variables were conducted with chi-square tests, two independent samples t-tests and Mann-Whitney U test, as appropriate. Multiple logistic regression analysis used the “*enter*” method (i.e., all specified independent variables were entered at one time) to determine the independent relationships between disability at 5 years follow up and variables that significantly differed in the above univariate analysis. The level of significance was set at 0.05 (two-tailed).

## Results

**Fig 1:**
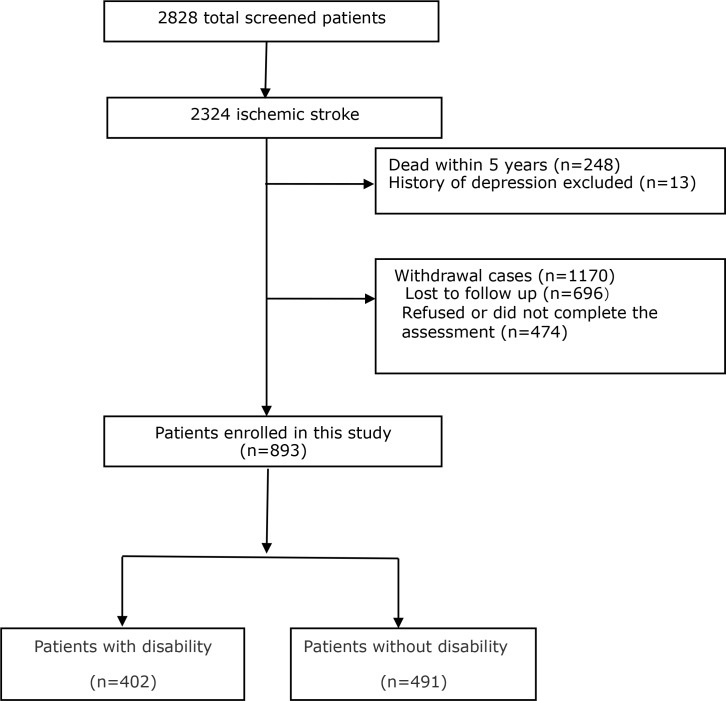
Flow diagram of the recruitment.

Out of 2,324 ischemic stroke patients who fulfilled study entry criteria at baseline, 248 died, 696 could not be located via the information recorded in medical records, and 474 refused or did not complete the telephone interview at the 5-year assessment. Finally, data on 893 patients were included in the statistical analyses (Fig 1). Compared to patients who completed the 5-year assessment, those who were excluded for any reasons were older and more likely to be single marital status (S1 Table). In addition, there were missing data in several variables (cardiac disease, current smoking and family history of stroke) with the percentages of less than 0.5%, except for hyperlipidemia (1.5%).

Altogether, 402 post-stroke patients (45.0%) met the criteria of disability. Table 1 shows the basic demographic and clinical characteristics of the whole sample, together with comparative data between the groups with and without disability. Compared to the group without disability, the group with disability at 5 years follow up were more likely to be older, female gender, have diabetes, cardiac disease, previous history of stroke, cognitive impairment and depression at 3 months, and stroke recurrence within the past 5 years, and have higher NIHSS score at baseline, higher HRSD score at 3 months, lower MMSE score at 3 months, but lower education level and less current smoking and moderate/heavy drinking.

**Table 1:** Comparison between stroke patients with and without disability at 5-year follow up with respect to demographic and clinical characteristics.

	**Total sample (n = 893)**		**No-disability (n = 491)**		**Disability (n = 402)**		**Statistics**		
	**N**	**%**	**N**	**%**	**N**	**%**	**χ^2^**	**df**	**p**
Male	614	68.8	352	71.7	262	65.2	4.3	1	**0.03**
Married/ cohabited	842	94.3	469	95.5	373	92.8	3.0	1	0.08
High school or above	367	41.1	219	44.6	148	36.8	5.5	1	**0.01**
Diabetes	217	24.3	106	21.6	111	27.6	4.3	1	**0.03**
Hyperlipidemia	249	28.3	130	27.0	119	29.9	0.9	1	0.33
Hypertension	620	69.4	328	66.8	292	72.6	3.5	1	0.06
Cardiac disease	189	21.3	89	18.2	100	25.0	6.0	1	**0.01**
Current smoking	333	37.4	205	41.9	128	31.9	9.4	1	**0.002**
Moderate/Heavy-drinking	138	15.5	94	19.1	44	10.9	11.3	1	**0.001**
Stroke History	197	22.1	70	14.3	127	31.6	38.6	1	**0.001**
Stroke Family History	179	20.1	104	21.2	75	18.8	0.8	1	0.36
**Toast Classification**									
Large-Artery etiology	570	63.8	308	62.7	262	65.2	0.5	1	0.44
Cardio-embolism etiology	24	2.7	9	1.8	15	3.7	3.0	1	0.08
Small-vessels etiology	216	24.2	118	24.0	98	24.4	0.01	1	0.90
Other determined or undetermined etiology	41	4.6	27	5.5	14	3.5	2.0	1	0.15
**Neuro-imaging characteristics**									
Left lesions	328	36.7	180	36.7	148	36.8	0.002	1	0.96
Right lesions	335	37.5	192	39.1	143	35.6	1.1	1	0.27
Frontal lobe	114	12.8	63	12.8	51	12.7	0.004	1	0.94
Temporal lobe	77	8.6	42	8.6	35	8.7	0.007	1	0.93
Parietal lobe	87	9.7	51	10.4	36	9.0	0.5	1	0.47
Occipital lobe	63	7.1	34	6.9	29	7.2	0.02	1	0.86
Basal ganglia	433	48.5	240	48.9	193	48.0	0.06	1	0.79
Thalamus	84	9.4	49	10.0	35	8.7	0.4	1	0.51
Brainstem	186	20.8	102	20.8	84	20.9	0.002	1	0.96
Cerebellum	68	7.6	36	7.3	32	8.0	0.1	1	0.72
Cerebral Lesions	237	26.5	133	27.1	104	25.9	0.1	1	0.68
Sub-cortical Lesions	495	55.4	274	55.8	221	55.0	0.06	1	0.80
Infra-tentorial Lesions	232	26.0	128	26.1	104	25.9	0.005	1	0.94
Cognitive Impairment at 3 months	129	14.4	41	8.4	88	21.9	32.7	1	**<0.001**
Depression at 3 months	198	22.2	90	18.3	108	26.9	9.3	1	**0.002**
Stroke recurrence within 5-years	211	23.6	65	13.2	146	36.3	65.2	1	**<0.001**
	**Mean**	**SD**	**Mean**	**SD**	**Mean**	**SD**	**T / Z**	**df**	**p**
Age (yrs)	60.6	10.7	57.6	10.2	64.2	10.2	-9.5	891	**<0.001**
NIHSS score at admission	4.4	3.6	3.9	3.2	5.0	3.9	-4.0	—^a^	**<0.001**
HRSD total at 3 months	4.8	4.8	4.3	4.5	5.3	5.0	-3.2	—^a^	**0.001**
MMSE total at 3 months	27.2	3.6	28.1	2.4	26.2	4.4	-7.3	—^a^	**<0.001**

Bold values are p<0.05; a = Mann-Whitney U test; HRSD = Hamilton Rating Scale for Depression-17; MMSE = Mini Mental State Examination; NIHSS = National Institutes of Health Stroke Scale; Toast = Trial of Org 10172 in Acute Stroke Treatment

Table 2 shows the independent correlates of disability in stroke patients. Older age, low education level, previous history of stroke, more severe stroke at baseline, greater depression and cognitive impairment at 3 months, and stroke recurrence within the past 5 years were significantly associated with disability (R^2^ = 0.47; p<0.001).

**Table 2:** Independent correlates of disability at 5 years in stroke patients (multiple logistic regression analysis with the group without disability as reference group).

	Stroke patients*		
Variables	p	OR	95% CI
Age	**<0.001**	1.06	1.04,1.08
Male	0.20	0.7	0.4,1.1
High school or above	**0.02**	0.6	0.4,0.9
Diabetes	0.75	1.06	0.7,1.6
Cardiac Disease	0.69	1.09	0.7,1.6
Current smoking	0.09	1.4	0.9,2.2
Moderate/Heavy Drinking	0.65	1.1	0.6,1.9
Stroke History	**<0.001**	2.6	1.7,4.1
NIHSS score at admission	**<0.001**	1.1	1.05,1.1
Depression at 3 months	**0.009**	1.8	1.1,2.9
Cognitive impairment at 3 months	**<0.001**	2.7	1.6,4.7
Stroke Recurrence within 5 years	**<0.001**	4.1	2.7,6.3

Bold values are p<0.05

* adjusting for study site; NIHSS = National Institutes of Health Stroke Scale

## Discussion

To the best of our knowledge, this is the first national large-scale longitudinal study in China to examine the disability rate of 5-year post-stroke and its associated factors.

In this study, 45% of post-stroke patients had disability, which is consistent with the range (36%-71%) reported in other Western surveys [21–23]. Variation between studies may partly be attributed to differences in definitions of disability (e.g. Barthel Index vs. mRS), length of follow up, post-stroke illness stage and sample size. In this study, those who dropped out were older than those who participated. Given the impact of advanced age on post-stroke disability, the prevalence rate in our study could be an under-estimation.

In this study cognitive impairment at 3-month post-stroke significantly contributed to the disability at 5-year follow up, which is consistent with earlier findings [18, 24, 25]. One study found that cognitive impairment, as defined by the MMSE score ≤24 at 3 months after the index stroke, independently predicted functional outcomes as measured by the Frenchay Activities Index up to 3 years in stroke survivors [18]. The mechanism for the association between cognitive impairment and poor functional outcome is unclear [26, 27]. Cognitive impairment was observed in multiple cognitive domains, especially in executive function, attention and processing speed, both of which are closely associated with physical function [24, 28]. Of note, both comprehensive neuropsychological instruments (e.g., the digit span test and frontal assessment battery) and brief screening tests (e.g., the MMSE or Montreal Cognitive Assessment) on cognition could detect vascular cognitive impairment [29].

The association between post-stroke depression and clinical outcomes in previous studies have been mixed[30]. Similar to some studies [31, 32], but not others [18, 33], we found that depression at 3 months post-stroke was highly predictive of disability at 5-year follow up. A recent study involving 1,101 stroke survivors in south London registry also found that depression at 3 months after stroke was predictive for disability up to 5 years follow up [32]. Depression causes both behavioral (e.g. decreased motivation or poor medication adherence) and biological changes (e.g. dysregulation in autonomic system activation, hypothalamic-pituitary-adrenocortial axis and inflammation states), which could increase the risk of disability [2, 32]. Post-stroke depression is likely to have a recurrent and chronic pattern which may partly explain the impact of depression after stroke on long-term disability [34].

Consistent with previous findings [35–37], older age, more severe stroke, previous history of stroke and stroke recurrence are found to be significant risk factors for long-term disability. Previous history of stroke and stroke recurrence may cause a culmination of deficits related to gait and impaired motor function arising from other brain lesions [2, 38]. Higher education was correlated with less disability, which is consistent with a study reported in India [39]. Well-educated patients may have less stigma, be more conscious about outcomes of stroke, and more likely to adhere to medications, and actively participate in physical rehabilitation [30], which could reduce the risk for long-term disability.

The major merits of this study include the large, multi-site, nationwide sample, the long-term 5-year outcome evaluation and the use of standardized assessments. However, there are several limitations in this study. First, only 38.4% of the baseline eligible patients completed the 5-year assessment. Due to the rapid urbanization and huge population migration in China, the high dropout rate in large scale cohort studies was unavoidable. Second, the mRS may not be sensitive enough to detect subtle changes in physical functioning. However, its contents are clinically distinct and responsive to patient-centered outcomes in stroke patients [40]. Third, it should be noted that the MMSE is not a stroke-specific cognitive measure. Fourth, several important variables, such as poststroke rehabilitation therapy, treatment adherence and the size of infarcts, white matter lesions and brain atrophy, were not evaluated at the 5-year assessment due to unavailable data. In addition, due to significant missing data of medication used, this was not included for analyses. Finally, patients who could not complete a clinical interview were excluded at baseline, which could lead to potential selection bias. Despite these methodological shortcomings, the findings are likely to approximate the general long-term profile of stroke patients across China.

In conclusion, long-term disability is highly prevalent in Chinese post-stroke patients. Neuropsychiatric (depression and cognitive impairment) and sociodemographic (older age and lower educational level) factors, poor stroke management (previous stroke history and recurrence stroke) and previous stroke severity are negatively associated with functional outcome. Adequate treatment of depression, secondary prevention of strokes and appropriate rehabilitation strategy need to be considered to reduce disability in post-stroke patients in China.

## Supporting Information

S1 Table Comparison of baseline characteristics between study completers and non-completers at 5-year. (DOC)
