# Supplementary material for: The Disability Rate of 5-Year Post-Stroke and Its Correlation Factors: A National Survey in China
Source: PLoS One. 2016 Nov 8;11(11):e0165341. doi: 10.1371/journal.pone.0165341 (PMC5100878; doi:10.1371/journal.pone.0165341)
Supplement: S1 Table — (DOC) [file pone.0165341.s001.doc]

**S1 Table.** Comparison of baseline characteristics between study completers and non-completers at 5-year

|  | **Withdrawl**  **(n=1170)** | | **Enrolled**  **(n=893)** | | **Statistics** | | |
| --- | --- | --- | --- | --- | --- | --- | --- |
|  | **N** | **%** | **N** | **%** | **χ2** | **df** | **p** |
| Male | 772 | 66.0 | 614 | 68.8 | 1.7 | 1 | 0.18 |
| Married/ cohabited | 1076 | 92.0 | 842 | 94.3 | 4.1 | 1 | **0.04** |
| High school or above | 449 | 38.5 | 367 | 41.1 | 1.3 | 1 | 0.24 |
| Diabetes | 313 | 27.2 | 217 | 24.3 | 2.1 | 1 | 0.14 |
| Hyperlipidemia | 245 | 22.6 | 249 | 28.3 | 8.3 | 1 | **0.004** |
| Hypertension | 762 | 66.0 | 620 | 69.4 | 2.6 | 1 | 0.10 |
| Cardiac disease | 281 | 24.2 | 189 | 21.3 | 2.5 | 1 | 0.11 |
| Current smoking | 369 | 31.8 | 333 | 37.4 | 6.9 | 1 | **0.008** |
| Moderate/Heavy-drinking | 151 | 12.9 | 138 | 15.5 | 2.7 | 1 | 0.09 |
| Previous stroke history | 291 | 24.9 | 197 | 22.1 | 2.2 | 1 | 0.13 |
| Stroke family history | 172 | 15.1 | 179 | 20.1 | 8.6 | 1 | **0.003** |
| **Toast Classification** | | | | | | | |
| Large-Artery etiology | 670 | 57.3 | 570 | 63.8 | 9.1 | 1 | **0.003** |
| Cardioembolism etiology | 31 | 2.6 | 24 | 2.7 | 0.003 | 1 | 0.95 |
| Small-vessels etiology | 291 | 24.9 | 216 | 24.2 | 0.12 | 1 | 0.72 |
| Other determined or undertermined etiology | 73 | 6.2 | 41 | 4.6 | 2.6 | 1 | 0.10 |
| **Neuroimaging characteristics** | | | | | | | |
| Left lesions | 410 | 35.0 | 328 | 36.7 | 0.6 | 1 | 0.42 |
| Right lesions | 443 | 37.9 | 335 | 37.5 | 0.02 | 1 | 0.87 |
| Frontal lobe | 136 | 11.6 | 114 | 12.8 | 0.6 | 1 | 0.43 |
| Temporal lobe | 122 | 10.4 | 77 | 8.6 | 1.8 | 1 | 0.16 |
| Parietal lobe | 134 | 11.5 | 87 | 9.7 | 1.5 | 1 | 0.21 |
| Occipital lobe | 87 | 7.4 | 63 | 7.1 | 0.1 | 1 | 0.74 |
| Basal ganglia | 602 | 51.5 | 433 | 48.5 | 1.7 | 1 | 0.18 |
| Thalamus | 106 | 9.1 | 84 | 9.4 | 0.07 | 1 | 0.78 |
| Brainstem | 240 | 20.5 | 186 | 20.8 | 0.03 | 1 | 0.86 |
| Cerebellum | 83 | 7.1 | 68 | 7.6 | 0.20 | 1 | 0.65 |
| Cerebral Lesions | 304 | 26.0 | 237 | 26.5 | 0.08 | 1 | 0.77 |
| Sub-cortical Lesions | 677 | 57.9 | 495 | 55.4 | 1.2 | 1 | 0.26 |
| Infra-tentorial Lesions | 300 | 25.6 | 232 | 26.0 | 0.03 | 1 | 0.86 |
|  | **Mean** | **SD** | **Mean** | **SD** | **T / Z** | **df** | **p** |
| Age (yrs) | 61.9 | 11.5 | 60.6 | 10.7 | 2.6 | 2061 | **0.009** |
| NIHSS score at admission | 4.5 | 3.5 | 4.4 | 3.6 | -6.03 | ---^a^ | 0.54 |

Bold values are p<0.05; a = Mann-Whitney U test; NIHSS = National Institutes of Health Stroke Scale; Toast=Trial of Org 10172 in Acute Stroke Treatment.
